# Posterior atrophy predicts time to dementia in patients with amyloid-positive mild cognitive impairment

**DOI:** 10.1186/s13195-017-0326-y

**Published:** 2017-12-16

**Authors:** Jung-Min Pyun, Young Ho Park, Hang-Rai Kim, Jeewon Suh, Min Ju Kang, Beom Joon Kim, Young Chul Youn, Jae-Won Jang, SangYun Kim, the Alzheimer’s Disease Neuroimaging Initiative

**Affiliations:** 1 Department of Neurology, Seoul National University Bundang Hospital, 82, Gumi-ro 173 Beon-gil, Bundang-gu, Seongnam-si, Gyeonggi-do 13620 Republic of Korea; 2 Department of Neurology, Seoul National University College of Medicine, Seoul, Republic of Korea; 3 Graduate School of Medical Science & Engineering, KAIST, Daejeon, Republic of Korea; 4 Department of Neurology, Kangwon National University Hospital, 156, Baengnyeong, Chuncheon, Kangwon 24341 Republic of Korea; 5 Department of Neurology, Chung-Ang University Hospital, Seoul, Republic of Korea

**Keywords:** Posterior atrophy, Biomarkers, Disease progression, Mild cognitive impairment, Alzheimer’s disease

## Abstract

**Background:**

In patients with amyloid-positive mild cognitive impairment (MCI), neurodegenerative biomarkers such as medial temporal lobe atrophy (MTA) are useful to predict disease progression to dementia. Although posterior atrophy (PA) is a well-known neurodegenerative biomarker of Alzheimer’s disease, little is known about PA as a predictor in patients with amyloid-positive MCI.

**Methods:**

We included 258 patients with amyloid-positive MCI with at least one follow-up visit, and who had low cerebrospinal fluid (CSF) β-amyloid_1–42_ concentration. Data were obtained from the Alzheimer’s Disease Neuroimaging Initiative study. We assessed PA and MTA on magnetic resonance imaging (MRI) using visual rating scales and retrospectively determined progression to dementia during the follow-up period of up to 3 years (median 24 months). The Cox proportional hazards model was used to analyze hazard ratios (HRs) of PA and MTA for disease progression. Additionally, subjects were divided into four groups according to brain atrophy pattern (no atrophy, MTA only, PA only, both MTA and PA), and HRs for disease progression were compared with the no atrophy reference group. Analyses were conducted with and without adjustment for CSF phosphorylated tau_181p_ (p-tau) and baseline demographics.

**Results:**

A total of 123 patients (47.7%) showed MTA and 174 patients (67.4%) showed PA. Of the total cohort, 139 cases (53.9%) progressed to dementia. PA and MTA were associated with an increased risk for progression to dementia (HR 2.244, 95% confidence interval (CI) 1.497–3.364, and HR 1.682, 95% CI 1.203–2.352, respectively). In the analysis according to atrophy pattern, HR (95% CI) for progression was 2.998 (1.443–6.227) in the MTA only group, 3.126 (1.666–5.864) in the PA only group, and 3.814 (2.045–7.110) in both MTA and PA group. These results remained significant after adjustment.

**Conclusions:**

In patients with amyloid-positive MCI, PA could predict progression to dementia independently of MTA.

**Electronic supplementary material:**

The online version of this article (doi:10.1186/s13195-017-0326-y) contains supplementary material, which is available to authorized users.

## Background

Dyshomeostasis of β-amyloid (Aβ) is an initiating factor of Alzheimer’s disease (AD) pathology [1]. Aβ positivity, defined as Aβ deposition on positron emission tomography (PET) or a low level of cerebrospinal fluid (CSF) β-amyloid_1–42_ (Aβ_1–42_), is known to predict clinical progression from mild cognitive impairment (MCI) to dementia [2, 3]. However, using only Aβ positivity for the prediction of disease progression is limited by the fact that Aβ positivity in CSF begins at least 15 years before expected clinical symptom onset [4]. Therefore, neurodegenerative biomarkers that reflect sequential pathologic processes after Aβ positivity could be useful in predicting progression from MCI to dementia in the near future [5, 6].

Neurodegeneration is the process of neuronal injury, and can be evaluated by atrophy observed on structural magnetic resonance imaging (MRI), hypometabolism on [^18^F]-fluorodeoxyglucose-PET, positive tau PET, or increased CSF tau [6–8]. Medial temporal lobe atrophy (MTA) on MRI is one of the most well-known neurodegenerative markers [9], and can predict progression from MCI to dementia [10, 11]. In a study with MCI patients with Aβ positivity, those with MTA were more likely to progress to dementia, which indicates the predictive value of MTA in amyloid-positive MCI [5].

Recently, an increasing number of studies have suggested that another neurodegenerative marker, posterior atrophy (PA) on MRI, could also predict conversion to dementia in MCI [11, 12]. In particular, evidence has shown that, whereas MTA is related to low levels of CSF Aβ_1–42_, PA is associated with high levels of CSF total tau (t-tau) and phosphorylated tau_181p_ (p-tau); this might support a prognostic value of PA in terms of disease progression among patients with amyloid-positive MCI [11]. The current study therefore aimed to assess the predictive value of PA for progression to dementia in amyloid-positive MCI.

## Methods

### Subjects

Data used in the preparation of this article were obtained from the Alzheimer’s Disease Neuroimaging Initiative (ADNI) database (adni.loni.usc.edu). The ADNI was launched in 2003 as a public-private partnership, led by Principal Investigator Michael W. Weiner, MD. The primary goal of ADNI has been to test whether serial MRI, PET, other biological markers, and clinical and neuropsychological assessments can be combined to measure the progression of MCI and early AD. For up-to-date information, see www.adni-info.org.

Data used in this study were downloaded from the ADNI database on 25 May, 2017. We included patients with late MCI who had had a baseline MRI scan, amyloid positivity on a CSF study [13], and at least one or more follow-up visits after initial assessment. The primary outcome of this study was progression to dementia during the follow-up period of up to 3 years. A final total of 258 patients, 143 from the ADNI1 cohort and 115 from the ADNI2 cohort, were included in this study.

Diagnosis of late MCI was made according to the presence of objective memory impairment but without meeting the criteria for dementia. Namely, all subjects had a Mini Mental State Examination (MMSE) score of 24 or higher, a global Clinical Dementia Rating (CDR) score of 0.5, a CDR memory score of 0.5 or higher, and a score indicating impairment on the delayed recall of Story A of the Wechsler Memory Scale-Revised (≥16 years of education: ≤ 8; 8–15 years of education: ≤ 4; 0–7 years of education: ≤ 2), which is 1.5 standard deviations below the mean score of cognitively normal subjects.

### CSF measurements and cutoffs

Baseline CSF Aβ_1–42_, t-tau, and p-tau were measured centrally using the multiplex xMAP Luminex platform (Luminex Corp, Austin, TX, USA) with Innogenetics (INNO-BIA AlzBio3, Ghent, Belgium; for research use-only reagents) immunoassay kit-based reagents, as described by Shaw and colleagues [13]. Qualification of the analytical performance of CSF samples from ADNI was controlled, showing within-center coefficient of variation (%CV) 95% confidence interval (CI) value (mean) of 4.0–6.0% (5.3%) for Aβ_1–42_, 6.4–6.8% (6.7%) for t-tau, and 5.5–18.0% (10.8%) for p-tau [14]. Inter-center %CV 95% CI ranged from 15.9 to 19.8% (17.9%) for Aβ_1–42_, 9.6–15.2% (13.1%) for t-tau, and 11.3–18.2% (14.6%) for p-tau [14]. Amyloid positivity was defined as CSF Aβ_1–42_ of less than 192 pg/ml [13].

### MRI

Brain MRI scans were acquired as previously described [15]. Participants in the ADNI1 cohort were scanned using either a 1.5 T or 3 T MRI scanner. All subjects in the ADNI2 cohort were scanned using a 3 T MRI scanner.

#### MTA scale

MTA was evaluated using a five-point rating scale developed by Scheltens et al. [16]. According to the height of the hippocampal formation and the width of the choroidal fissure and the temporal horn, atrophy was rated from 0 (no atrophy) to 4 (severe atrophy) (Additional file 1: Table S1). The largest vertical height of hippocampal formation was defined as dentate gyrus, hippocampus proper, subiculum, and parahippocampal gyrus.

#### PA scale

PA was assessed using a four-point rating scale developed by Koedam et al. (0 = no atrophy; 1 = mild widening of the sulci without evident volume loss of gyri; 2 = substantial widening of the sulci and volume loss of the gyri; 3 = severe end-stage atrophy) [17]. The evaluated anatomical regions included the posterior cingulate sulcus, precuneus, parieto-occipital sulcus, and the cortex of the parietal lobes. MRI scans were assessed in the three different orientations by following anatomical landmarks: widening of the posterior cingulate and parieto-occipital sulcus and atrophy of the precuneus in the sagittal orientation, widening of the posterior cingulate sulcus and the sulcal dilatation in the parietal lobes in the axial orientation, and widening of the posterior cingulate sulcus and parietal lobes in the coronal orientation. In case of different rating scores between different MRI planes, the higher score was used for analysis.

#### Image analysis

All MRI scans were evaluated by three raters (board-certified neurologists, Young Ho Park, Hang-Rai Kim, Jeewon Suh, with 7, 5, and 4 years of experience in dementia) who were blinded to the clinical information. In case of disagreement, the three raters reviewed the MRI scans together for adjustment.

Intra-rater reliability was assessed by the re-rating of 25 randomly determined MRI scans at a separate sitting, blinded to their own prior rating. Inter-, and intra-rater reliabilities were measured by calculating the intraclass correlation coefficient.

For rating scores of both MTA and PA, scores of the right and left hemispheres for each patient were summed and the mean value was used. These scores were dichotomized as normal (no atrophy) or abnormal (atrophy). For MTA in those younger than 75 years old a rating score of 1.5 or more was considered abnormal, and in those aged 75 years or older a score of 2 or more was considered abnormal [18]. For PA, a score of 1.5 or more was considered abnormal in all patients [17].

For quantitative analysis we used data downloaded from the ADNI database. Regional volumes were measured automatically by the Freesurfer image analysis suite, which is freely available for download (http://surfer.nmr.mgh.harvard.edu/). ADNI1 1.5 T data were run with Freesurfer version 4.3, and ADNI1 3 T data and ADNI2 data were run with Freesurfer version 5.1. Each scan was segmented according to an atlas defined by Freesurfer [19]. We compared volumes of the hippocampus, parahippocampal cortex, entorhinal cortex, and fusiform gyrus in temporal regions as well as the superior and inferior parietal cortex, precuneus, supramarginal gyrus, and postcentral gyrus in parietal regions between groups with and without progression to dementia, using the Mann–Whitney test or Student’s *t* test as appropriate.

### Statistical analysis

For comparison of demographic and clinical variables between groups with and without progression to dementia, we used the Pearson chi-squared test, Mann–Whitney test, or Student’s *t* test as appropriate. We assessed the hazard ratio (HR) of MTA, PA, CSF t-tau, p-tau, baseline demographics, and neuropsychological profiles using univariate Cox regression analysis with follow-up time as a time variable and progression to dementia as a status variable. Additionally, we categorized MRI atrophy pattern into the following four groups: no atrophy, MTA only, PA only, and both MTA and PA. The HR of each group for disease progression was calculated using univariate Cox analysis. The proportional assumption was examined by log-log survival plots.

The multivariate Cox analysis was performed to identify independent determinants of disease progression with relevant covariates. The clinically or statistically relevant covariates with a *p* value < 0.2 in univariate Cox regression analysis were included. If there was more than one variable that was clinically highly correlated, we included only one of them in the model. Also, according to the atrophy pattern, two different models were implemented. In model 1, HRs of the MTA group and PA group were analyzed with adjustment for clinically or statistically relevant covariates. In model 2, HRs of the four groups according to atrophy pattern (no atrophy, MTA only, PA only, both MTA and PA) were analyzed with adjustment for relevant covariates. Multicollinearity between the covariates was tested by calculating the variance inflation factor [20]. We used SPSS 21 (SPSS Inc., Chicago, Illinois, USA) for multicollinearity and inter- and intra-rater reliabilities analyses, and R (version 3.3.1; http://www.R-project.org) for the remainder of the analyses. Demographic analysis was performed using wilcox.test, chisq.test, or t.test function, and Cox regression analysis was performed using the Coxph function in R with survival package version 2.41-3.

## Results

A total of 258 patients participated in the study. The median age of patients was 74.1 years, and 101 (39.1%) were female. A total of 176 patients (68.2%) had at least one *APOE* ε4 allele. Of the total cohort, 123 patients (47.7%) showed MTA and 174 patients (67.4%) showed PA. Dividing patients according to atrophy pattern, we identified 51 patients (19.8%) with no atrophy, 33 patients (12.8%) with MTA only, 84 patients (32.6%) with PA only, and 90 patients (34.9%) with both MTA and PA. During the follow-up period (median 24 months) 139 patients (53.9%) progressed to dementia, and 119 patients did not. Demographic, cognitive, and biomarker characteristics according to progression to dementia, categorized as stable MCI and progressive MCI, are summarized in Table 1. Patients with disease progression to dementia had poorer cognitive performances at baseline, higher levels of CSF t-tau and CSF p-tau, and more frequent MTA and PA compared with those without progression. The inter-rater reliability for MTA (0.84–0.87) and PA (0.70–0.87) was good. The intra-rater reliability for MTA (0.83–0.98) was excellent and for PA (0.73–0.98) was good (Additional file 2: Table S2). The volumes of the hippocampus, entorhinal cortex, fusiform gyrus, superior and inferior parietal cortex, precuneus, and supramarginal gyrus were significantly decreased in progressive MCI as compared to stable MCI (Additional file 3: Table S3).

**Table 1:** Baseline characteristics of the study sample

	All (*n* = 258)	Stable MCI (*n* = 119)	Progressive MCI (*n* = 139)	*p* value^c^
Age, years	74.1 (69.5–78.5)	74.4 (69.1–78.2)	73.6 (69.8–78.7)	> 0.999
Female, *n* (%)	101 (39.1)	40 (33.6)	61 (43.9)	0.119
Education, years	16 (14–18)	16 (14–18)	16 (14–18)	0.469
*APOE* ε4 carrier, *n* (%)	176 (68.2)	78 (65.6)	98 (70.5)	0.473
Cognition				
MMSE	27 (25–29)	28 (26–29)	26 (25–28)	< 0.001
CDR SB	1.5 (1.0–2.5)	1.5 (1.0–2.0)	2.0 (1.0–2.5)	< 0.001
ADAS-cog 11	11.8 (9.0–15.7)	10.7 (7.2–13.2)	13.0 (10.8–16.2)	< 0.001
CSF markers				
Aβ_1–42_, mean ± SD, pg/mL	136.1 ± 25.7	136.7 ± 27.2	133.7 ± 25.0	0.345
CSF t-tau, pg/mL^a^	104.0 (77.0–148.5)	91.0 (68.0–133.0)	113.0 (84.0–153.0)	0.006
CSF p-tau, pg/mL^b^	41.0 (31.0–58.0)	37.0 (27.0–51.0)	45.0 (36.0–64.0)	< 0.001
MRI				
Tesla, *n* (%)				0.714
1.5	143 (55.4)	64 (53.8)	79 (56.8)	
3.0	115 (44.6)	55 (46.2)	60 (43.2)	
MTA, *n* (%)	123 (47.7)	47 (39.5)	76 (54.7)	0.021
PA, *n* (%)	174 (67.4)	65 (54.6)	109 (78.4)	<0.001
Atrophy pattern, *n* (%)				<0.001
No atrophy	51 (19.8)	39 (32.8)	12 (8.6)	
MTA only	33 (12.8)	15 (12.6)	18 (13.0)	
PA only	84 (32.6)	33 (27.7)	51 (36.7)	
Both MTA and PA	90 (34.9)	32 (26.9)	58 (41.7)	

Data are presented as the median (interquartile range) unless otherwise specified

^a^ Data for 7 subjects were not available

^b^ Data for 1 subject were not available

^c^ Stable MCI vs. progressive MCI

*Aβ* β-amyloid, *ADAS-cog* Alzheimer’s Disease Assessment Scale-cognitive subscale, *CDR SB* Clinical Dementia Rating Sum of Boxes, *CSF* cerebrospinal fluid, *MCI* mild cognitive impairment, *MMSE* Mini-Mental State Examination, *MRI* magnetic resonance imaging, *MTA* medial temporal lobe atrophy, *PA* posterior atrophy, *p-tau* tau phosphorylated at threonine 181, *SD* standard deviation, *t-tau* total tau

**Fig. 1:**
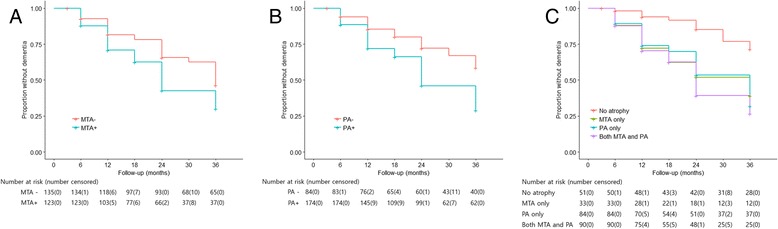
Cox proportional hazards model for progression to dementia in amyloid-positive mild cognitive impairment patients according to brain atrophy on MRI. PA (**a**), MTA (**b**), and atrophy pattern (no atrophy, MTA only, PA only, both MTA and PA) (**c**). *MTA* medial temporal lobe atrophy, *PA* posterior atrophy

In the univariate Cox regression analysis, the presence of MTA and PA showed significantly increased HR (95% CI) of progression to dementia with a value of 1.682 (1.203–2.352) and 2.244 (1.497–3.364), respectively (Fig. 1). In the analysis according to MRI atrophy pattern, patients with only MTA, only PA, and both MTA and PA were associated with a higher risk for disease progression compared with those with no atrophy (Table 2). Higher levels of CSF t-tau and CSF p-tau were related with more disease progression. Baseline cognitive performances with lower MMSE scores, higher CDR Sum of Boxes (CDR SB), and higher Alzheimer’s Disease Assessment Scale-cognitive subscale 11 (ADAS-cog 11) scores were also associated with progression to dementia. The proportional assumption was satisfied for MTA and PA based on log-log survival plots (Additional file 4: Figure S1).

**Table 2:** Univariate and multivariate Cox regression analysis

	Univariate HR (95% Cl)	Multivariate	
		Model 1 HR (95% Cl)	Model 2 HR (95% Cl)
Age	0.999 (0.976–1.022)	0.996 (0.970–1.023)	0.998 (0.972–1.025)
Female	1.266 (0.906–1.770)	1.152 (0.797–1.665)	1.162 (0.8031–1.682)
Education	0.961 (0.907–1.019)	0.954 (0.900–1.011)	0.961 (0.906–1.020)
*APOE* ε4 carrier	1.290 (0.896–1.858)	0.996 (0.674–1.470)	1.031 (0.700–1.520)
Cognition			
MMSE	0.821 (0.748–0.901)^b^	NI	NI
CDR SB	1.593 (1.347–1.883)^b^	1.526 (1.276–1.824)^b^	1.531 (1.279–1.832)^b^
ADAS-cog 11	1.118 (1.080–1.157)^b^	1.101 (1.061–1.144)^b^	1.104 (1.063–1.147)^b^
CSF markers			
CSF t-tau*	1.003 (1.000–1.006)^a^	NI	NI
CSF p-tau**	1.012 (1.006–1.019)^b^	1.006 (1.000–1.013)	1.006 (1.000–1.013)
MRI			
MTA	1.682 (1.203–2.352)^a^	1.424 (0.997–2.034)^a^	NI
PA	2.244 (1.497–3.364)^b^	1.895 (1.239–2.897)^b^	NI
Atrophy pattern			
No atrophy	Reference	NI	Reference
MTA only	2.998 (1.443–6.227)^a^	NI	3.178 (1.520–6.645)^b^
PA only	3.126 (1.666–5.864)^b^	NI	3.209 (1.693–6.080)^b^
Both MTA and PA	3.814 (2.045–7.110)^b^	NI	3.598 (1.909–6.783)^b^

* Data for 7 subjects were not available

** Data for 1 subject were not available

^a^ *p* < 0.05

^b^ *p* < 0.001

Model 1: adjusted for MTA, PA, age, sex, education, *APOE* ε4 carrier, ADAS-cog 11, CDR SB, and CSF p-tau

Model 2: adjusted for MRI atrophy pattern (no atrophy, MTA only, PA only, both MTA and PA), age, sex, education, *APOE* ε4 carrier, ADAS-cog 11, CDR SB, and CSF p-tau

*ADAS-cog* Alzheimer’s Disease Assessment Scale-cognitive subscale, *CDR SB* Clinical Dementia Rating Sum of Boxes, *CI* confidence interval, *CSF* cerebrospinal fluid, *HR* hazard ratio, *MMSE* Mini Mental State Examination, *MRI* magnetic resonance imaging, *MTA* medial temporal lobe atrophy, *NI* not included, *PA* posterior atrophy, *p-tau* tau phosphorylated at threonine 181, *t-tau* total tau

Multivariate Cox analysis included clinically (age, sex) and statistically relevant variables (education duration, *APOE* ε4 allele, ADAS-cog 11, CDR-SB, and CSF p-tau) (Table 2). Although MMSE and CSF t-tau were statistically relevant and variance inflation factors were less than 1.719 for all variables, indicating a low degree of collinearity, we excluded them from the multivariate Cox analysis because they were clinically highly correlated with ADAS-cog 11 and CSF p-tau, respectively. The adjusted covariates did not alter the significance of the HRs (95% CI) of MTA (1.424 (0.997–2.034)) or PA (1.895 (1.239–2.897)). Moreover, the HR of groups with only MTA, only PA, and both MTA and PA remained significant. Notably, there was little difference between the HRs of MTA only, PA only, and both MTA and PA. On the contrary, the significant relationships between CSF p-tau and disease progression disappeared after adjustment with other covariates.

## Discussion

The ability to predict progression from MCI to dementia is increasingly important with the prospect of disease-modifying therapies. This study demonstrated that patients with amyloid-positive MCI with PA or MTA are more likely to progress to dementia. Furthermore, patients with amyloid-positive MCI with PA only (in the absence of MTA) also showed an increased risk for disease progression. This indicates that PA, as well as MTA, is a predictive marker of conversion from amyloid-positive MCI to dementia.

To understand the pathophysiology of AD, considerable effort has been made to identify AD-related focal regions or functional connectivity between regions in the brain [21–24]. According to pathological staging by Braak and Braak, the neurofibrillary changes (tau pathology) start from the medial temporal lobe and spread to neocortical association areas [25]. Many neuroimaging studies have demonstrated these pathological changes, showing low glucose metabolism and cortical atrophy, most prominently in the medial temporal lobe and parietal lobe, including the bilateral precuneus, posterior cingulate cortex, and angular gyrus [21, 24, 26]. MTA within these regions is widely recognized as an imaging marker of AD [16]. Additionally, the parietal lobe has been highlighted as an area involved in the pathological changes and dysfunction in AD [27].

The parietal cortex is originally known for its visuospatial and sensorimotor functions [23]. However, the parietal cortex is also involved in other functions, such as episodic memory retrieval [23, 28]; notably, episodic memory retrieval impairment is a typical feature of AD. Functional MRI (fMRI) studies with memory-related tasks have revealed that the parietal cortex, especially the precuneus and superior and inferior parietal lobules, is involved in memory retrieval [29–31]. Furthermore, resting-state fMRI studies have revealed that patients with MCI display lower levels of neuronal activity in the posterior cingulate cortex, precuneus, and inferior parietal lobe (components of default mode network) compared with healthy controls [32, 33]. Concerning cortical connectivity, these regions comprise the “posterior medial network” (PM network), along with the retrosplenial cortex and parahippocampal cortex [34]. The PM network and anterior temporal network are two largely segregated pathways with different anatomical regions and different memory-guided behaviors, and were proposed by Ranganath and Ritchey [34]. The PM network is involved in episodic memory, spatial navigation, and scene perception. Dominant disruption of this network has been observed in patients with AD compared with healthy participants and patients with other types of dementia [21, 22, 34].

Systematic assessment of PA using a visual rating scale was suggested by Koedam and colleagues [17]. This visual rating scale, used to rate MRI images for PA within the posterior cingulate gyrus, precuneus, and parietal lobe, is simple, easily implemented in clinical settings, and has a good discrimination ability between healthy individuals and patients with AD [17]. The visual rating scale has been validated using voxel-based morphometry (VBM), with good reliability [35]. Furthermore, in a study of patients with pathologically proven definite AD, 30% of patients showed only PA without MTA, which indicates that PA could be used as an independent imaging marker of AD [36].

From the perspective of cognitive reserve, MCI patients with both MTA and PA are expected to progress more rapidly to dementia than patients with MTA only or patients with PA only [37]. However, progression rates were not different between patients with both MTA and PA, patients with MTA only, and patients with PA only in our study. Similar results have been reported regarding clinical progression of patients with AD [38, 39]. In those studies, which grouped patients with AD into three subtypes according to cortical atrophy patterns, patients with diffuse atrophy did not show more rapid progression than patients with medial temporal dominant atrophy or parietal dominant atrophy. Rather, patients with parietal dominant atrophy showed a faster progression rate [38, 39]. Although we do not know the reason, MTA and PA might not have additive effects on clinical progression in patients with amyloid-positive MCI.

With respect to VBM analysis in previous studies with patients with MCI, gray matter differences between patients with progressive and stable MCI were assessed using two-sided *t* tests in early studies [40]. However, the two-sided *t* tests discard information about varying lengths of follow-up times among patients. To overcome this problem, time-to-event statistical methods were used in VBM analysis of patients with MCI [41]. These studies revealed that the patients with progressive MCI showed volume loss in the medial temporal lobes as well as the temporoparietal cortex and frontal lobes compared with patients with stable MCI [40–42]. A recent study, which used a VBM survival analysis and assessed the effects of amyloid deposition on progression to dementia in patients with MCI, found that the pattern of decreased gray matter volume that was predictive of progression was similar in amyloid-positive and amyloid-negative patients [42]. Although our study demonstrated the usefulness of visually assessed PA for predicting progression to dementia in patients with amyloid-positive MCI, visually assessed PA might also be useful in patients with amyloid-negative MCI. In our previous study of patients with MCI without information about amyloid positivity, visual rating of PA had predictive value for progression to dementia [12].

Notably, our population showed a relatively large proportion of *APOE* ε4 carriers (62%). This might be due to characteristics of our amyloid-positive population. Other studies with amyloid-positive MCI also reported a large percentage of *APOE* ε4 carriers [5, 43, 44]. A considerably higher prevalence of amyloid positivity has been previously reported among *APOE* ε4 carriers compared to *APOE* ε4 noncarriers [45]. The relationship between *APOE* ε4 and amyloid positivity has been investigated extensively for its important pathological role and contributing risk to AD. *APOE* ε4 is known to increase AD risk by decreasing Aβ clearance and promoting Aβ aggregation [46]. Apolipoprotein E4 has lower affinity to Aβ than apolipoprotein E3, thus showing inefficient removal of Aβ across the blood-brain barrier and increased oligomerization of Aβ [47–49]. This aspect could support the large proportion of *APOE* ε4 carriers of our amyloid-positive MCI population.

The significant association found between increased CSF p-tau levels and disease progression in our study is consistent with previous studies [50]. However, this significance disappeared in multivariate analysis, contrary to PA or MTA. This could indicate that brain atrophy might correlate more strongly with clinical progression than CSF biomarkers. Several studies have shown similar correlations between MRI, CSF, and cognitive performance [51, 52]. One possible explanation for the stronger relationship of brain atrophy with progression (compared to the relationship of CSF biomarkers with progression) is that MRI may be a more stable biomarker for neuronal injury than CSF tau proteins, which can be influenced by diurnal variation and transient brain injury [51, 52]. Otherwise, disease progression with brain atrophy could be affected by factors other than tauopathy, such as aging, traumatic brain injury, toxic factors, and vascular factors [53–56].

There are some limitations to our study that should be noted. First, we defined amyloid positivity by CSF Aβ_1–42_ levels only, and did not include a population with positive amyloid PET. This may have led to a selection bias. However, several studies have shown there to be a good agreement between CSF Aβ_1–42_ levels and amyloid PET, which could minimize the potential bias in our study [57, 58]. Second, the visual rating scale may not be precise compared with volumetric quantitative measurements. Although we compared volumetric measures of temporal and parietal regions between stable and progressive MCI, we could not perform voxel- or surface-based analysis because our sample comprised ADNI1 and ADNI2 cohorts with different magnetic field strength.

## Conclusions

This study showed that PA in amyloid-positive MCI is significantly associated with disease progression to dementia, independent of the presence of MTA. This is indicative of the predictive value of PA for disease progression in patients with amyloid-positive MCI.

## Additional files

Additional file 1: Table S1. Visual rating of medial temporal lobe atrophy. (DOCX 14 kb)

Additional file 2: Table S2. Inter- and intra-rater reliability. (DOCX 16 kb)

Additional file 3: Table S3. Comparison of volumetric measures of temporal and parietal regions according to disease progression to dementia. (DOCX 19 kb)

Additional file 4: Figure S1. Log-log survival plots of PA (A) and MTA (B). *MTA* medial temporal lobe atrophy, *PA* posterior atrophy. (TIF 83 kb)

## Abbreviations

Aβ: β-Amyloid
AD: Alzheimer’s disease
ADAS-cog: Alzheimer’s Disease Assessment Scale-cognitive subscale
ADNI: Alzheimer’s Disease Neuroimaging Initiative
CDR: Clinical Dementia Rating
CDR SB: Clinical Dementia Rating Sum of Boxes
CI: Confidence interval
CSF: Cerebrospinal fluid
CV: Coefficient of variation
fMRI: Functional magnetic resonance imaging
HR: Hazard ratio
MCI: Mild cognitive impairment
MMSE: Mini Mental State Examination
MRI: Magnetic resonance imaging
MTA: Medial temporal lobe atrophy
PA: Posterior atrophy
PET: Positron emission tomography
PM: Posterior medial
p-tau: Phosphorylated tau_181p_
t-tau: Total tau
VBM: Voxel-based morphometry
