# Supplementary material for: Posterior atrophy predicts time to dementia in patients with amyloid-positive mild cognitive impairment
Source: Alzheimers Res Ther. 2017 Dec 16;9:99. doi: 10.1186/s13195-017-0326-y (PMC5732486; doi:10.1186/s13195-017-0326-y)
Supplement: Supplementary file 1 — Visual rating of medial temporal lobe atrophy. (DOCX 14 kb) [file 13195_2017_326_MOESM1_ESM.docx]

**Table S1.** Visual rating of medial temporal lobe atrophy.

| Score | Width of  choroid fissure | Width of  temporal horn | Height of  hippocampal formation |
| --- | --- | --- | --- |
| 0 | N | N | N |
| 1 | ↑ | N | N |
| 2 | ↑↑ | ↑ | ↓ |
| 3 | ↑↑↑ | ↑↑ | ↓↓ |
| 4 | ↑↑↑ | ↑↑↑ | ↓↓↓ |

↑ = increase, ↓ = decrease, N = normal.
