# Supplementary material for: Posterior atrophy predicts time to dementia in patients with amyloid-positive mild cognitive impairment
Source: Alzheimers Res Ther. 2017 Dec 16;9:99. doi: 10.1186/s13195-017-0326-y (PMC5732486; doi:10.1186/s13195-017-0326-y)
Supplement: Supplementary file 2 — Inter- and intra-rater reliability. (DOCX 16 kb) [file 13195_2017_326_MOESM2_ESM.docx]

**Table S2.** Inter- and intra-rater reliability.

|  | Rater | MTA | PA |
| --- | --- | --- | --- |
| Inter-rater reliability | 1 and 2 | 0.87 (0.83–0.90) | 0.77 (0.71–0.83) |
|  | 2 and 3 | 0.84 (0.79–0.87) | 0.70 (0.62–0.77) |
|  | 1 and 3 | 0.84 (0.80–0.88) | 0.87 (0.84–0.90) |
| Intra-rater reliability | 1 | 0.98 (0.94–0.99) | 0.95 (0.89–0.98) |
|  | 2 | 0.83 (0.62–0.93) | 0.73 (0.39–0.88) |
|  | 3 | 0.96 (0.92–0.98) | 0.98 (0.94–0.99) |

Values are presented as the ICC (95% CI).

Rater 1: Young Ho Park, Rater 2: Hang-Rai Kim, Rater 3: Jeewon Suh

*CI* confidence interval, *ICC* intraclass correlation coefficient, *MTA* medial temporal lobe atrophy, *PA* posterior atrophy
