# Supplementary material for: Posterior atrophy predicts time to dementia in patients with amyloid-positive mild cognitive impairment
Source: Alzheimers Res Ther. 2017 Dec 16;9:99. doi: 10.1186/s13195-017-0326-y (PMC5732486; doi:10.1186/s13195-017-0326-y)
Supplement: Supplementary file 3 — Comparison of volumetric measures of temporal and parietal regions according to disease progression to dementia. (DOCX 19 kb) [file 13195_2017_326_MOESM3_ESM.docx]

**Table S3.** Comparison of volumetric measures of temporal and parietal regions according to disease progression to dementia.

| **Region** | **Stable MCI**  **(n = 119)** | **Progressive MCI**  **(n = 139)** | **p value^*^** | |
| --- | --- | --- | --- | --- |
| **Temporal lobe** | | | | |
| R parahippocampal | 1835.0 (1599.5–2027.0) | 1797.0 (1630.5–1992.0) | 0.573 |  |
| L parahippocampal | 1954.5 ± 383.3 | 1892.6 ± 312.6 | 0.161 | |
| R entorhinal | 1668.0 (1463.0–1981.0) | 1477.0 (1288.5–1738.5) | < 0.001 |  |
| L entorhinal | 1782.7 ± 427.0 | 1578.1 ± 410.8 | < 0.001 | |
| R fusiform | 8534.0 (7563.5–9503.0) | 8043.0 (7173.5–8913.5) | 0.001 |  |
| L fusiform | 8828.9 ± 1432.1 | 8324.7 ± 1359.7 | 0.004 | |
| R hippocampus | 3326.2 ± 567.2 | 3041.3 ± 485.2 | < 0.001 | |
| L hippocampus | 3267.2 ± 545.4 | 2991.3 ± 454.9 | < 0.001 | |
| **Parietal lobe** | | | | |
| R postcentral | 7989.5 ± 1156.9 | 7865.8 ± 1250.3 | 0.413 | |
| L postcentral | 8379.0 (7682.5–9005.5) | 8124.0 (7511.5–9179.5) | 0.614 |  |
| R precuneus | 8241.0 (7414.0–9920.5) | 7917.0 (7111.0–8872.5) | 0.030 |  |
| L precuneus | 8227.9 ± 1263.9 | 7751.0 ± 1130.5 | 0.002 | |
| R superior parietal | 11276.3 ± 1623.3 | 10889.1 ± 1882.8 | 0.081 | |
| L superior parietal | 11423.5 ± 1825.0 | 10870.4 ± 1809.4 | 0.015 | |
| R supramarginal | 9029.6 ± 1494.2 | 8633.9 ± 1474.1 | 0.034 | |
| L supramarginal | 9481.1 ± 1485.3 | 8915.9 ± 1490.0 | 0.003 | |
| R inferior parietal | 12829.0 ± 2014.5 | 12038.3 ± 2032.3 | 0.002 | |
| L inferior parietal | 10342.0 (9311.0–11967.5) | 9867.0 (8625.0–10882.5) | 0.005 |  |

Data are presented as the median (interquartile range) or mean ± standard deviation.

^*^ For statistical analysis we used the Mann–Whitney test or Student’s *t* test as appropriate.

*R* right, *L* left
