# Supplementary figures and images for: Posterior atrophy predicts time to dementia in patients with amyloid-positive mild cognitive impairment
Source: Alzheimers Res Ther. 2017 Dec 16;9:99. doi: 10.1186/s13195-017-0326-y (PMC5732486; doi:10.1186/s13195-017-0326-y)

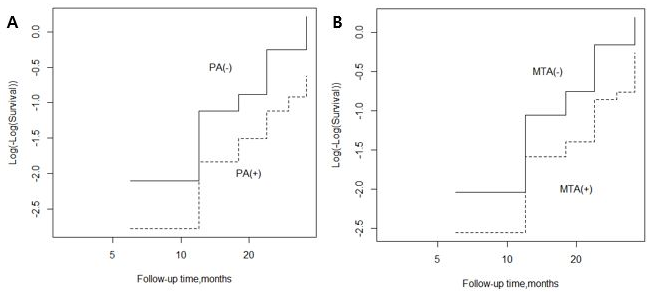

Supplement: Supplementary file 4 — Log-log survival plots of PA (A) and MTA (B). MTA medial temporal lobe atrophy, PA posterior atrophy. (TIF 83 kb) [file 13195_2017_326_MOESM4_ESM.tif]
